# Epistemic appropriation and the ethics of engaging with trans community knowledge in the context of mental healthcare research

**DOI:** 10.1186/s13010-024-00157-9

**Published:** 2024-05-21

**Authors:** Francis Myerscough, Lydia Schneider-Reuter, Mirjam Faissner

**Affiliations:** 1 Phoenix Song Project, Bristol, UK; 2 Institute for Medical Ethics and History of Medicine, Ruhr University Bochum, Bochum, Germany; 3 Department of Psychiatry, Psychotherapy and Preventive Medicine, LWL University Hospital, Ruhr University Bochum, Bochum, Germany; 4 Institute of the History of Medicine and Ethics in Medicine, Charité - Universitätsmedizin Berlin, Berlin, Germany

**Keywords:** Epistemic injustice, Gender diversity, Trans rights, Structural oppression, Research ethics

## Abstract

Mental healthcare research increasingly focuses the needs of trans people and, in doing so, acknowledges knowledge and epistemic resources developed in trans communities. In this article, we aim to raise awareness of an ethical issue described by Emmalon Davis that may arise in the context of engaging with community knowledge and epistemic resources: the risk of epistemic appropriation. It is composed of two harms (1) a detachment of epistemic resources developed in the originating community and (2) a misdirection of these epistemic resources for epistemic goals of a dominant community. In this article, we map and discuss the ethical concerns in using knowledge originating in trans communities in terms of epistemic appropriation in the context of mental healthcare research. We first argue that misgendering, failing to reference non-academic sources and a lack of attribution in community authorship are forms of epistemic *detachment*. Second, we problematize cases of epistemic *misdirection* of trans epistemic resources, focusing on the examples of detransition and transition regret. We discuss harms related to epistemic appropriation in relationship to risks to safety. The article aims to raise awareness about the risk of epistemic appropriation both in researchers engaging with trans knowledge as well as in mental healthcare workers who seek information on trans.

## Introduction

In recent years, mental healthcare research has increasingly turned towards questions concerning healthcare for trans individuals, e.g. regarding the access to treatments [1, 2], diagnostic categories [3], therapy practice and therapist competencies [4–7].^1^ This is a timely development given that trans people have high mental health burdens due to minority stress and structural discrimination [7]. Gender-affirmative care is effective in addressing the mental health burden of trans individuals and is therefore considered an important part of trans healthcare [6–8]. Additionally, in light of the current internationally observable, increasingly hostile social and political climate around trans rights [9–11], research aiming at improving trans healthcare is welcome.

Knowledge production by and about trans people is a diverse practice that has led to heterogeneous bodies of knowledge: besides the medical-psychiatric discourse on gender diversity, trans community structures are a site of knowledge production. Due to the ongoing structural discrimination and the continuing pathologizing of trans through psychiatric institutions throughout the 20th century [8], people who might nowadays call themselves trans or gender non-conforming gathered in private, counter-public and sub-cultural spaces [12]. For many, these spaces have served as places of chosen kinship, community support, understanding and mutual care. Within these spaces, people have developed knowledge and conceptual resources, such as language, concepts and narratives, about trans life and experiences. As Stryker [12] shows, it is community efforts and knowledge production that eventually achieved positive changes in the ways trans subjects were represented, discussed and supported internationally, for example in media, law, psychiatry, and healthcare. Eventually, academic fields such as trans studies [13–16], trans philosophy [17–19], and trans epistemology [20] have emerged. Still today, trans community knowledge production and distribution is an important part of trans activism and care work. It can be found in the realm of academia, for example in a book edited by Laura Erickson-Schrot [21] that features articles written by trans people for trans people, as well as online. Here, tools such as blogs can function as sites of resistance with informational, testimonial and activist goals [22]. Therefore, gender-affirming care today can be understood as encompassing various practices, including services offered in formal healthcare settings, community-based support structures, and self-care practices.

With a rise of trans-positive academic research, trans community knowledge is increasingly recognized and sought in mental healthcare research as well. While this may be judged as an overall positive development, there are also different risks that arise for trans communities if mental healthcare researchers draw on trans community knowledge. Accordingly, a growing literature has started to address ethical aspects of producing and evaluating trans centered academic works [23–25], with specific regard to trans health research [26, 27]. The mentioned literature addresses many important aspects that promise to improve trans healthcare research, especially when doing empirical research.

In this article, we aim at contributing to the discussion by showing how mental healthcare research may (also unintentionally) contribute to the epistemic appropriation of trans community knowledges. We first introduce ethical problems that arise, if trans knowledge is *detached* from trans communities and trans researchers through problematic citation practices, misgendering and a lack of attribution in community authorship. We then problematize cases of epistemic *misdirection* of trans epistemic resources and knowledge, focusing on detransition and transition regret. We sketch further harms connected to the risk of safety to trans communities in publishing trans community knowledge and suggest that these different forms of harm need to be considered alongside each other. Eventually, we discuss some implications for mental healthcare researchers and for clinicians who aim to acquire competencies for trans-affirmative care.

Our epistemic stance is informed by our social positioning, as one White^2^, cis, female, abled, queer person, one White, trans and queer, abled person and one White, trans and queer, disabled person. Academic backgrounds involve medicine, music therapy, philosophy and gender studies. Methodologically, we base our analysis on the philosophical literature from the field of trans philosophy and combine it with examples from trans mental healthcare research. As part of anchoring our thinking to research practice, lived experience, and to acknowledge and exemplify the complexities of ethical questions in the area, we draw on one author, Francis Myerscough’s, work with the co-produced music therapy organization for and by trans people, Phoenix Song Project. Phoenix Song also serves as an example of how valuable community knowledge (shared in media such as blogs, books, videos, and social media [28] and gathered through personal experiences in trans communities) is in clinical practice, especially since little academic knowledge had been published when the project was launched in 2019.

## Epistemic appropriation

### Background– epistemic appropriation and trans epistemic communities

Scholars working on epistemic injustice, i.e. forms of injustice that occur in the context of knowledge sharing and production within systems of power and oppression [29–31], have paid special attention to ethical problems that arise if knowers from dominant social positions draw on epistemic resources from epistemically marginalized social groups [32, 33]. Dominant social positions are those which have access to more social, material, and political power than other, marginalized, social positions. In the context of mental healthcare research, academic researchers, clinicians, psychologists and psychiatrist are often positioned on dominant positions due to their institutional roles. Additionally, many of them are also positioned in socially dominant positions as cis, hetero, and White.^3^ Emmalon Davis explains that marginalized communities (i.e., those which possess less material, political and social power) may develop epistemic resources (such as concepts, theories, narratives, metaphors, tropes) which are apt to describe and interpret their own social experiences and lived reality. As long as these epistemic resources are shared *within* their own subcultures, we can call them *intracommunal*. Under some circumstances, epistemic resources may be spread and used in other epistemic communities; they then become available *intercommunally*.^4^ For instance, use of the term ‘cisgender’, its shortened form ‘cis’ and related concepts such as ‘cis-sexism’ in relation to gender were developed in the 1990ies in trans communities [20].^5^ Since the 1990ies, the usage of ‘cis’ and related terms in relation to gender has been widely adopted by researchers, scholars and activists outside trans communities.

According to Davis, when intracommunal epistemic resources acquire uptake beyond the original epistemic community, there is the risk of a particular epistemic injustice: the risk of ‘epistemic appropriation’ [33]. Epistemic appropriation is characterized by two harms. First, the epistemic resource is *detached* from the original individuals who have developed the epistemic resource, so that the originator is not publicly recognized. A second harm in epistemic appropriation is the epistemic *misdirection* of the epistemic resource. In this case, the epistemic resource is used to serve epistemic goals other than those of the marginalized epistemic community. Made up of these two, often interwoven, harmful processes, epistemic appropriation is oppressive insofar as it undermines marginalized groups in their epistemic agency.^6^ In the next part, we introduce problems that arise, if trans knowledge is *detached* from trans communities and trans researchers in misgendering and a lack of attribution in community authorship, before problematizing cases of epistemic misdirection of trans epistemic resources within mental healthcare research.

### Detachment of trans community knowledge and epistemic resources in mental healthcare research

Epistemic resources and bodies of knowledge can be detached from their originators if they are *not* or not *correctly* attributed to those who have primarily produced and developed them [33]. In the context of research produced by trans scholars and activists, three main problems arise that involve detachment: misgendering, failing to reference non-academic sources and failing to acknowledge community authorship.^7^

Misgendering and ‘deadnaming’ constitutes a form of detachment through inconsiderate citation practices when citing trans scholars that holds specific power over trans people. Deadnaming happens if authors are referred to by a name which they do not use – typically a name assigned to them at birth. Misgendering is mostly associated with the use of wrong pronouns. This is not always deliberate, but also happens inadvertedly if bibliographic technologies are not efficiently updated.^8^ Stephanie Kapusta notes that as pronouns and information on gender identity might not always be available, the potential harm of misgendering for trans scholars – including psychological, moral and political harms – should *always* be considered when making attributions [37]. Misgendering may not only manifest through the use of pronouns or designations associated with gender assignments, but also through definitions of gender terms that exclude parts of the trans community, including non-binary people, from it [37–39].^9^ The risk of misgendering is not only relevant to citation practices, but is also present when undertaking empirical research with trans people. For example, in some empirical publications, authors misgendered trans women as men who have sex with men [24]; while these authors may have been seeking to reflect people’s anatomical features in their research, this could have been captured by the use of anatomical language. Furthermore, there are clinical implications to the misidentification of trans women as men who have sex with men: to misidentify populations in this way firstly obscures the differences in sexual practices and discourses between members of each community. While there are overlaps, there are also separate conversations and sexual practices within trans communities. For example, Mira Bellwether’s *Fucking Trans Women* [40] is an influential sex education zine written by a trans woman for trans women, and the people who have sex with them. Secondly, should such language erasing trans women filter into the clinical practice of services, then trans women are less likely to feel welcome and safe enough to access these services, or simply to understand from the language that the services are not for them.

Detachment through misgendering is particularly relevant for (mental) healthcare research due to its symbolic meaning and its effect. Sarah Cavar and Alexandre Baril [41] highlight that especially disabled trans people face barriers in being seen as legitimate by medical authorities.^10^ Misgendering trans authors, especially trans scholars with disabilities who face intersecting forms of oppression, may reproduce trans-exclusive norms. Additionally, misgendering may signal to trans scholars that they are not welcome to participate in institutional knowledge production. Given psychiatry’s own historical contribution to pathologizing trans people, for instance through the inclusion of “Transsexuality” as a mental disorder in the influential *Diagnostical and Statistical Manual of Mental Disorders*-III [45], it is important for psychiatry to provide a welcoming research environment for trans scholars [8].

Other challenges with regards to epistemic detachment arise when scholars quote non-academic sources. This is particularly relevant in the context of trans knowledge, as much knowledge on trans, such as DIY HRT (i.e. self-medicating or sourcing hormones oneself, without a prescription) or knowledge on how to get access to certain medical procedures, is developed by trans people through their own experience [46]. For example, when writing a reflective article [47] about experiences of (in)visibility and (in)audibility as a member of minoritized communities, Francis included references to non-academic sources such as threads on Twitter and Reddit. There was theory being shared on Twitter that captured perspectives they had never encountered formalized in an academic context – namely, The Tweedy Mutant’s [48] reframing of so-called invisible disability reflecting not something inherent to the disability or disabled person, but others’ bodymind literacy, or lack thereof: does the other person know what the visual or sonic signs of a disability are? Has society equipped them with the resources to perceive disability? Francis was unsure how the author would want to be referenced; this was someone who they knew was undertaking a research degree, but his Twitter did not contain his name, only the pseudonym *The Tweedy Mutant*. On this occasion Francis was able to make contact and check if and how this author would want references to appear, but this is likely not to be the case in all situations. Here, a dilemma arises between acknowledgement of the person who has developed the knowledge on the one hand and increased, potentially unwanted visibility for the person cited on the other. Nonetheless, even in the case of anonymizing trans community knowledge in order to increase safety (be it one’s own safety as an author or the safety of the person whose knowledge is being cited), the harmful detachment of community knowledge might be avoided by at least consciously giving credit to the concerned community.^11^

Instances of epistemic detachment may also arise if academic researchers use epistemic resources that have not been developed by singular authors, but *intracommunally* – meaning that the resources stem from dialogues taking place between individuals within marginalized communities, as in DIY HRT. While in the situation above, Francis was easily able to identify an individual to cite, in the form of the person’s twitter account, this is more difficult when understandings have developed out of dialogue and discussion: Jessica Collier and Corrina Eastwood [51] have highlighted how present understandings of intersectionality are drawn from grassroots practice in addition to the critical lens put forward by Kimberlé Crenshaw [52]. Reflecting on their engagement with critical theory more broadly, Francis and their colleague Denise Wong [28] noted how they came to be familiar with critical theoretical frameworks often through informal conversations or interactions within their respective minoritized communities, rather than through an academic setting. When this is the case, it may be difficult or impossible to identify clearly defined sources, or what sources can be identified may be defined in a manner which does not fully align with academic expectations. If an identifiable source cannot be defined, one path may be to cite a source which corroborates the claim being made, but was not the source of the claim for the writer(s). However, would such a path be an accurate portrayal of background for the text, and for how the writer(s) came to this claim? When there is an identifiable source, but one which is defined in a differing manner to academic convention, a path of adaptation (and possibly also explanation) is an option. For example, Sabah Choudrey [53] makes reference to “wise words of the Queensland Aboriginal activist group (1970s)” (p.185), and includes a footnote regarding this source, and the lack of more specific dating. In short, this is a dilemma about how to cite the origin of knowledge which has not come from academic settings, and has sources which do necessarily find an easy fit in academic citation practices.

Misgendering, failing to reference non-academic sources and failing to acknowledge community authorship are thus all different forms of epistemic detachment that especially concern trans scholars and communities. Davis [33] suggests that through detachment a person’s “status as an epistemic contributor is consequently unrecognized”. As Miranda Fricker [54] notes, being recognized as a subject of knowledge, and in Davis’ terms, as a contributor of knowledge, is an aspect central to humanity. Since trans people have for a long time been pathologized and epistemically and politically marginalized, not acknowledging the origin of knowledges in trans communities seems especially problematic and harmful.

### Epistemic misdirection of trans community knowledge in mental healthcare research

Davis [33] introduced the term ‘epistemic misdirection’ to describe cases in which “the benefits associated with the epistemic contributions of the subordinate disproportionately benefit the powerful.” The debate on the epistemic misdirection of trans knowledge by researchers has a long history: In his “Suggested Rules for Non-Transsexuals Writing about Transsexuals, Transsexuality, Transsexualism, or Trans __”, published in 1997, Jacob Hale challenges researchers to interrogate their own motivations when writing with reference to trans people [55]. Hale suggests that writing on trans is only morally permissible if it accepts trans existence and does not question its validity; the minimal working hypothesis preceding every writing on trans should be: “Transsexual lives are lived, hence livable.” Hale urges researchers to interrogate their own subject position, specifically in regard to trans, to reflect the ways in which one has access to power compared to trans people, how this affects one’s research practices and knowledge production, as well as the interests and stakes that are forming one’s initial interest.^12^ One reason for these proposed high standards of writing about trans lies in the high risk of epistemic misdirection of trans knowledge. For example, Viviane Namaste [19] argues that in Anglo-American feminist theory, referring to trans experiences is often aimed at answering the fields’ *own* epistemological questions (meaning in many cases the questions of cis women) instead of questions relevant to the epistemic interests of trans people, pointing to a potential instrumentalization of the ‘transgender question’.^13^

Misdirection of trans community knowledge is also a challenge for mental healthcare research, for example in research on so-called ‘detransition’ and transition regret. Broadly speaking, where transition refers to the social, medical and/or administrative changes trans people make or go through in an attempt to live an affirming life, detransition refers to the phenomenon of individuals deciding to stop or reverse some or all of these changes, whether partially or completely, temporarily or indefinitely. Transition regret is a distinct phenomenon from detransition but may contribute to a person’s decision to detransition. The discourse on detransition is complex and heterogeneous. Much of the knowledge about detransition and transition regret is developed and shared within the trans community, for instance on social media, such as reddit and tiktok. At the same time, ideas about detransition are also much discussed in anti-trans contexts, e.g. in trans exclusionary radical feminist groups.^14^

As Rowan Hildebrand-Chupp [62] points out, a significant amount of emerging research on the wide spectrum of detransition experiences focuses on the causes of detransition and detransition rates. This research implies that detransition necessarily is a negative clinical outcome that needs to be prevented. Such interpretation and stance on regret reflects a lack of the necessary “conceptual know-how”, defined by Fricker and Katharine Jenkins as a “range of conceptual competences requisite for understanding a sphere of social experience had by the in-group”, to understand the contributions of trans communities [25]. As noted above, within trans communities, specific sets of concepts and meanings have been developed to make sense of trans experiences. For instance, the concept of “detransgender” or “detrans” as used in trans subcultural practices differs from dominant ideas about detransition experiences. For people who have detransitioned, being detrans may in fact be neutral or positive and may present an integral and cherished part of their gender experience. Thus, the concept of detrans as used in trans (including detransition) communities involves different and more complex sets of value judgements, including positive ones. If healthcare researchers are unaware of socio-linguistic practices within trans culture, they may fail to properly understand contributions from trans communities or even the data they gathered from their study participants. Lack of conceptual know-how may therefore lead to epistemic distortions [20, 25]. As a consequence, the output of the research may not benefit trans communities since their research interests (e.g. medical effects of different ways of detransitioning) may not be sufficiently represented in research designs which are based on a misconception of “detrans”.

To acknowledge trans conceptual resources and to improve the conceptual know-how by healthcare researchers, Jack L. Turban et al. [63]– building on trans community knowledge– call on researchers to use precise language that does not conflate the idea of detransition with a cis identity, regret, or the delegitimization of an individual’s self-knowledge regarding their gender identity. Turban et al. [64] propose terms such as “discontinuation of gender-affirming medical care, regret regarding gender-affirming medical care, or evolution regarding conceptualization of one’s gender identity” and a framework that accounts for internal and external factors influencing these phenomenona. Such careful research practices and acquiring conceptual know-how are especially important since the fact that people chose to discontinue and reverse gender-affirming medical or surgical care has been politized and used as evidence to prohibit the provision of medical care [64]. Especially in the US and in recent years, negative transition experiences and transition regret by individuals have been used for trans-antagonistic political goals, impacting access to gender-affirming medical and surgical care especially for trans adolescents [65]. This indicates that epistemic misdirection leaves way for political instrumentalization.

A specific case of a political instrumentalization of negative transition experiences and transition regret, discussed within the trans community, can be found in the in the Bell v Tavistock court case.^15^ The 2020 UK High Court Ruling [66] in favour of Bell rested strongly on the argument that young people under the care of the Tavistock Gender Identity Development Service were not Gillick competent to give informed consent with respect to taking puberty blockers.^16^ The High Court ruling was overturned in the Court of Appeal in 2021 [68]. The focus on Gillick competence is significant, because Gillick is utilized as a benchmark of competence in many medical disciplines, and in particular with respect to contraceptive treatment, the area from which the term emerged. Research by journalists [69, 70] and community organizations [71] highlighted Bell’s lawyer, Paul Conrathe’s involvement in previous anti-abortion cases, along with other causes of Christian right wing groups. As such, the spotlight on Gillick in Bell v. Tavistock raised concerns not only for trans people, their allies and advocates, but also for the impact the judgement on Gillick might have in other areas concerned with children and young people’s autonomy, and particularly with regard to their access to contraception, including emergency contraception and abortion.^17^ While the initial high court ruling was later successfully appealed [73], the example shows that the instrumentalizing of the “transgender question” is a real concern beyond the academy and has the potential to harm individuals outside of trans communities as well. Cases such as Bell v Tavistock allow one to reflect on the complex relationship of anti-trans political movements, the epistemic misdirection of concepts developed in trans communities (such as detrans), the political instrumentalization of single or small groups of trans voices, mental healthcare research, and mental healthcare practice. Here, the possibly unintentional epistemic misdirection of trans knowledge and experiences, based on insufficient conceptual know-how, may provide a basis for political instrumentalization. There is thus a high risk that conceptual resources on detransition, rather than serving trans and detransition communities and their medical and mental health, may be used for anti-trans ideological goals. At the same time, it should be noted that there is no way to avoid false and hostile uptake of careful and thorough research on detransition experiences and transition regret. Yet, the mentioned legislative initiatives that impact the access to trans healthcare call for especially nuanced research that centers the needs of trans and detransition communities.

### Risks on safety for trans communities

So far, given the negative impact of epistemic appropriation on the epistemic agency of trans communities, one might assume that it follows that avoiding epistemic appropriation should be the primary goal of all researchers. Simultaneously, the context of continued and increasing hostility toward trans people calls for research practices that, in addition to the harms of epistemic appropriation, take into account risks to the safety of trans people on an individual and community level. As our discussion of citation practices indicates, the goal of avoiding detachment of community knowledge cannot be easily achieved without considering risks on safety that might follow from explicitly crediting trans individuals or communities. Rather than resolving the tensions between epistemically sound practices and safety, we aim to show that an epistemically-cum-ethically praiseworthy conduct necessitates taking into account both epistemic appropriation and safety.

Risk of harm is commonly understood as situational across a variety of disciplines [74–76]. In keeping with this, the levels of risk for harm posed through knowledge production towards given groups are variable in likelihood and severity dependent on the societal context(s) in which the knowledge production and the people risks are posed towards are situated. It is, therefore, necessary to consider the socio-political climate to understand why, how, and what kind of risks may be posed. In places that show a rise of the far right and fascism, as for example the UK [77, 78], where trans people increasingly experience the passing of hostile laws [9, 79] and a rise in hate crimes [79–81], the political context suggests a higher likelihood of adverse consequences both for trans people engaged with knowledge production (as authors, providing critique, or otherwise), and broadly for members of the trans community.

On the level of the trans community, publication of community practices also means knowledge of these is made available to anti-trans individuals and groups, who may use this information against the community. For example, publicly sharing information regarding DIY HRT may result in criminalization of individuals or groups, or of the systems trans people have put in place to meet healthcare needs being closed down or undermined through legislative, criminal means, or otherwise. At the same time, it is important that information is available such that people who belong to or support trans communities can learn and grow. Also, publishing this research means that there is a greater visibility of trans people. Greater visibility can increase the probability of being attacked for one’s (assumed) transness: while visibility of minoritized groups is widely celebrated as a good, Joli St. Patrick [82] reflects on the risks associated with it: “For many trans women, visibility is exactly the problem: it is involuntary, and it leaves us vulnerable to both physical and social violence. We get mocked, harassed, talked down to – and trans women of color [sic] get murdered on a regular basis.”

Working as a trans scholar, and being visible as a trans scholar, entails significant risks to individual safety. For example, to be named in a publication, or other public aspects of knowledge production such as presenting at conferences, is to make oneself more identifiable. Sometimes this is only by name, but often such attributions include further details such as a workplace or even a workplace address. Should such activity involve disclosure of trans identity – whether explicitly or implicitly– the inclusion of such details would make identification easier. For example, it may identify trans people to trans exclusionary activists who could target them, whether in person, or through online “doxxing” (whereby identifying details about a person are shared publicly online, frequently including contact details, often along with shaming and inflammatory commentary). For example, one author is aware of other trans scholars in their region being sent hate mail using their university addresses. Also, one author reflects on being hesitant of being openly trans in some academic settings and in publications due to the anticipation of hostile reactions. Even if the work of a trans person is cited favourably, there is the potential for backlash from hostile people and groups who might follow the citation. Moreover, even when this backlash does not occur, many trans people will nevertheless feel justifiably anxious that it might.^18^ This highlights that trans people, including trans scholars, find themselves in a particularly vulnerable position when engaging with knowledge production. While they can and do add especially valuable insight, they experience disproportionate burdens and risks in offering critique [83].^19^

## Implications

What follows from this for mental healthcare research on trans which engages with knowledge from trans communities? Different authors, such as Radi [20] and Namaste [19] have formulated positive principles for research on trans to ensure that it does not reproduce or support the epistemic marginalization of trans people, e.g.: developing “meticulous empirical research” [20], developing knowledge only in use for the community, giving decision power to trans people in trans research projects, and ensuring data property.

These principles are in line with our analysis from which we infer a special responsibility to consider who is addressed and whether the information published can be harmful to the community. Importantly, the property principle implies that trans communities have a right to preserve secrecy of trans knowledges. Establishing relationships with trans community members and asking which information can be published and which should be kept confidential is key to ethical research. This can happen in the form of collaborations in which all people involved in a project epistemically collaborate on *equal* footing. On the part of cis researchers, co-producing knowledge demands work in acquainting themselves with trans conceptual know-how as far as possible through using the already published and openly available resources.

On the institutional level, the risks described call for structures that are prepared to protect trans scholars and trans focused research in case of detachment, misdirection, instrumentalization, and attacks on safety. To account for this, journals, universities, and academic communities should be in contact with trans community stakeholders and may develop guidelines on epistemic collaboration with the goal of following them transparently and with the goal of accountability.

What does our analysis imply for mental healthcare staff who strive to learn more about trans in order to support trans service users and clients? We suggest that they need to be aware of how political and legal initiatives that affect access to gender affirming care may epistemically appropriate trans community knowledge. This is especially important since the politization and instrumentalization of detransition experiences might increase already existing fears that discourage people from addressing worries they might have and asking questions of their mental healthcare providers that relate to detransition [91]. Against this background, mental health staff might benefit from prioritizing the acquisition of conceptual know-how that allows them to identify political instrumentalization. Regular training programs on gender and sexual diversity are not institutionally established as part of standard curriculums for students as well as for practicing mental health staff. Hence, mental health staff need to be aware of their responsibility to educate themselves in order to provide adequate care for trans individuals as well as people who (want to) destransition. Online resources, such as the website *Health Liberation Now!* that offer information on transition and detransition experiences, may be of use here [61]. The conceptual know-how sought after by mental health staff should include intersectional perspectives that account for the experiences of multiply marginalized trans people as they experience disproportionate epistemic marginalization and are disproportionally affected by anti-trans movements.^20^Additionally, it should entail the acknowledgement of the potentials of community led support programs outside of the realm of formal psychiatry, and the ability to inform service users about such programs.^21^ This seems of relevance not only in contact with trans users, but may be beneficial for working with users in general.

## Conclusion

In this article we have shown different ways in which trans community knowledge is vulnerable to epistemic appropriation: Trans knowledge can be detached from their source via misgendering, deadnaming and not correctly referencing non-academic sources and community authorship. We also highlighted struggles of epistemic misdirection of trans knowledge, which may be epistemically misdirected to serve purposes of dominant groups.

Considering the existing literature cited within this article alongside our own thinking and argument, we note the extent to which certain points have endured over the course of decades, while others have not. For example, Hale’s [55] suggestion that researchers should interrogate their own subject position remains extremely relevant. On the other hand, changes in the political climate have reshaped some of the questions we believe researchers should be asking when doing research on trans; we specifically highlighted that the resurgence of far-right politics and the intercommunal sharing of trans community knowledge may contribute to risks faced by trans individuals and communities.

This positioning of knowledge production as part of the situation which contributes to or reduces risks – including risks of violence – to individuals and communities is significant. Acknowledging such a link underscores the importance and consequence of work in this area: this is not a theoretical exercise – it impacts people’s lives, including the potential for these lives to be taken away.

While the main issues we raised may apply to all academic fields, mental healthcare research seems to hold a particularly powerful position in regard to trans quality of life. Its potential to counteract dominant and pathologizing narratives on trans health can only be fulfilled if researchers are aware of the ways in which epistemic appropriation of trans community knowledge can take place and what might follow from it – or how it might enable the continuation of epistemic oppression. At the same time, epistemic appropriation of trans community knowledge in mental healthcare research can have detrimental effects on trans life, since clinicians’ approaches to trans, treatment plans, legal developments and societal attitudes may be influenced by misdirections of trans knowledge.

We acknowledge that there is not a quick and simple fix to the concerns raised in this article. As with the cultures we build around mistakes, accountability, and care, there is likely to be a complex web of – sometimes very small – adjustments to be made amounting to a significant overall shift; this is an ongoing process. Overall, and at every stage should the desired cultural shifts take place, it is crucial that authors reflect critically about their subject position towards trans people, and the human impact of their writing practices. We thus highlight the specific responsibility to reflect on risks and safety issues, and praiseworthy epistemic conduct when engaging with trans community knowledge. Epistemic exchange and mutual epistemic relationships between marginalized and dominant knowers can contribute to social change; and especially in current times hold the potential of meaningful, even life-saving impact.

## Data Availability

Not applicable.

## Footnotes

1 In this article, we use the term trans, also commonly referred to as ‘transgender’, as an umbrella term to refer to people whose gender identity and assigned gender at birth are not, or only partially aligned. Aiming for a broad conceptualization of trans, we understand trans to include people who identify as for example non-binary, agender, genderqueer or gender nonconforming. We use the term cis, or cisgender, when referring to people whose gender identity aligns with their assigned gender at birth.

2 We capitalize White in order to highlight that Whiteness is a social and epistemic position and in order to prevent Whiteness from being presented as the unquestioned norm.

3 Such hierarchies also exist *between* professional groups concerned with mental health, though exploring these positionings is outside the focus of the present article.

4 It should be added that we do not understand an epistemic community as bound together by simply sharing the same social identity or lived experience. Rather, it is created by the collective production, proximity to and sharing of certain epistemic resources.

5 Simply a Latin preposition meaning ‘on this side of’ [34] – the opposite of trans – ‘cis’ had ancient origins before being applied to gender.

6 We draw on Kristie Dotson’s [30] notion of epistemic oppression.

7 Blas Radi [20] describes the example of using knowledge on HIV prevalence developed by trans women without referencing them as “epistemic extractivism”, which we may call another case of epistemic detachment.

8 In a blog post, Amy Dobrowolsky exemplifies this with an experience in which library records outed a trans scholar by use of their former name [35]. The lack of awareness for a needed accommodation of bibliographical practices in contact with trans scholars calls to attention the power citation practices can hold and danger they can inflict when trans people are not considered in bibliographical and citational practices [36].

9 Katja Thieme and Mary Ann S. Saunders plead for a careful consideration in relation to disclosing trans scholars’ identity via integral citation, meaning when naming the cited author in a sentence rather than parentheses or footnotes [36]. Drawing from an analysis of articles in Transgender Studies Quarterly, they observe the direct naming of an author’s trans identity only in cases where said author had auto-ethnographically written about their trans experience, attesting to what they consider a potential scholarly community of care or ethics of care in which visibility and the dissemination of trans knowledge as well as considerations of trans peoples’ safety are equally sought after.

10 In part, this relates to the ‘adultification’ of transition [42], or, indeed, other explorations of gender that do not conform to cis-heteronormativity, combined with the infantilization of disabled people. For example, in relation to those labelled/with intellectual disability, the drag troupe of performers with Down Syndrome, Drag Syndrome, have been targeted multiple times by campaigns claiming that they are taking advantage of learning disabled people, who should be kept “innocent” [43]. At time intertwined with infantilization, disabled people’s experience, autonomy, and dignity is frequently disregarded, including in ways that relate to gender. For example, Jamie Hale [44] has exemplified the consistently inconsistent pronouns professionals used when referring to them in meetings relating to their funding package for care at home, as well as the disbelief expressed by some professionals that anyone like them would be able to consent to sex and have any intimate relationships.

11 Questions on ethical engagement with publicly shared social media content are increasingly being discussed with regards to power dynamics and marginalized communities (e.g. Matamoros-Fernández and Farkas 2021 [49]). Nonetheless, qualitative and quantitative social media research often lacks considerations of complex consent dynamics and potential risks to authors and referenced individuals [49, 50].

12 It should be noted that this aspect as well as the following are not to be considered by non-trans people exclusively. As entire research fields are shaped by certain narratives, commonsense or “emotional habitus” [56], it is equally trans *and* non-trans people who must reflect on their contribution to knowledge projects. Cameron Awkward-Rich exemplifies this powerfully, when pointing out how the founding narrative of trans studies followed the “strategy of securing trans authority through the disavowal of sick” [57], therefore further marginalizing trans sex workers, poor and disabled trans people and trans people of color. In another example, Cressida J. Heyes and J. R. Latham point to the politics of dis/analogy between gender affirming surgeries (for trans people) and cosmetic surgeries (for non-trans people) [58]. In analyzing the argument that differentiates gender affirming healthcare strongly from cosmetic surgeries by a degree of psychological suffering of trans people, they emphasize the ways in which this narrative puts trans people in a position in which the diversity of their experiences get undermined and they have to fear being disqualified from accessing care when diverging from the normative model of trans suffering and psychological assessment. An awareness of exclusionary effects and intersectional discrimination hence needs to be applied regardless of identity category and equally in contact with knowledge from trans communities.

13 Namaste quotes Judith Butler’s [59] work on violence against trans people used to understand conditions of survival and vulnerability of “the very category human” as an example of Anglo-American feminist theory that serves feminist theory more than trans people [19].

14 For instance, Ky Shevers draws on her own experience of detransitioning while engaged with such a trans exclusionary radical feminist community [60, 61]. Now disengaged from the community, Shevers reflects on this experience as a form of ideologically motivated ‘conversion practice’ and aims at raising awareness of possible harms [61].

15 This court case was brought against the Tavistock and Portman NHS trust by a young person who was previously under the care of the trust’s Gender Identity Development Service (GIDS), and later detransitioned. This was the only NHS GIDS service available for children and young people in England and Wales. We concentrate in our article on how this case was instrumentalized; one young person’s journey being instrumentalized against gender affirming care more widely, and the topic of gender-affirming healthcare being instrumentalized in a larger effort to undermine bodily autonomy.

16 Gillick competence has been defined as: the means by which to assess legal capacity in children under the age of 16 years, established in the case Gillick v West Norfolk and Wisbech Area Health Authority (1985) 2 A11 ER 402. Such children are deemed to be capable of giving valid consent to advice or treatment without parental knowledge or agreement provided they have sufficient understanding to fully appreciate the nature, purpose, and hazards of the proposed treatment. In the Gillick case the criteria for deciding competence, set out by Lord Fraser, related specifically to contraceptive treatment. In addition to the elements of Gillick competence, the Fraser guidelines specified that a health professional must be convinced that the child was likely to begin, or to continue having, sexual intercourse with or without contraceptive treatment, that the child’s physical and/or mental health would probably suffer in the absence of treatment, and that it was in the child’s best interests to provide treatment. The principle of Gillick competence applies to all treatment for those under the age of 16 [67] (pp. 774–775).

17 The observation of potential harm afflicted upon other marginalized groups (in this case children and adolescents as a whole) as an effect of anti-trans legislation and politics is not unique to Bell v Tavistock. As V. Jo Hsu [72] highlights, trans rights often “serve as a political battleground for much broader social issues” impacting especially the lives of people of color, disabled people, queer people, and the groups’ intersections. According to Hsu, this happens because anti-trans actors – driven by affect and while devaluing trans knowledges – build on racist, ableist and heteropatriarchal logics when attacking trans rights.

18 While physical attack or doxxing are relatively defined instances of targeting, there is also a slower-paced and ongoing burden of engaging in knowledge production as a trans person. Kapusta [83] has highlighted the burden to trans people of engaging in critique. The expectation of repeatedly justifying one’s existence, work, and worth is another burden further to those experienced by those with normalised identities. For example, one of the authors has found that, as a known trans person, people will often approach them with questions whenever there has been something relating to trans people in the news, and expect the author to be willing to engage in swift and patient education, regardless of whatever else the author is doing at the time. More specific to knowledge production, trans and nonbinary people may feel the need to advocate for themselves in the face of knowledges which are trans exclusionary, with the potential for discussion to escalate to more explicit denial or demonisation of their communities. The heightened anxiety and vigilance many trans people develop in order to anticipate and be more prepared for experiences of discrimination and oppression such as these is a further drain on these thinkers’ energy and resources; therein there is not only a potential to undermine effectiveness in knowledge reproduction roles, but also health and wellbeing, since hypervigilance is a trauma response, and psychological trauma is connected with a number of chronic illness conditions [84–86]. This is in keeping with the connections being made between minority stress and chronic health conditions for various minoritized communities [87–90].

19 This is not to say that people who do not consider themselves part of the trans community do not experience risk: there is no “zero-risk” scenario, and the consideration above of how “the trans question” is instrumentalized in separate arguments, and in parallels outside the academy, exemplifies how this can take place. Functional and fair accountability processes (or lack thereof) are also a matter of relevance for all, regardless of gender-identity history.

20 This entails a critical awareness of the dominance of White knowledge-producers in trans community spaces [22] as well as among trans scholars [53].

21 For instance, the project LETS (https://projectlets.org/) develops alternative mental health support structures, based on peer-led community-based approaches to healing.
